# Blood and cerebrospinal fluid characteristics in neonates with a suspected central nervous system infection

**DOI:** 10.1097/MD.0000000000016079

**Published:** 2019-06-21

**Authors:** Dirkje de Blauw, AHL Bruning, LJ Vijn, JG Wildenbeest, KC Wolthers, MH Biezeveld, Anne-Marie van Wermeskerken, Femke Nauta, Dasja Pajkrt

**Affiliations:** a Amsterdam University Medical Centers, location Academic Medical Center (AMC), Department of Pediatric Infectious Diseases Amsterdam, The Netherlands; b Amsterdam University Medical Centers, location Academic Medical Center (AMC), Department of Medical Microbiology, Amsterdam, The Netherlands; c Department of Paediatric Infectious Diseases, University Medical Center Utrecht, The Netherlands; d Department of Paediatrics, Onze Lieve Vrouwe Gasthuis. Amsterdam, The Netherlands; e Department of Paediatrics, Flevohospital, Almere, The Netherlands.

**Keywords:** blood characteristics, CNS infections, CSF characteristics, neonates

## Abstract

Clinical signs and symptoms of central nervous system (CNS) infections in neonates are often nonspecific. Therefore, cerebrospinal fluid (CSF) analysis is performed to diagnose CNS infections. Data on combined microbiological results and their correlation with biochemical characteristics in CSF and blood in infants younger than 90 days are limited. This study provides an overview of microbiological test results, CSF- and hematological characteristics among infants with a clinically suspected CNS infection.

This retrospective study included infants younger than 90 days, with a clinically suspected CNS infection who underwent a diagnostic lumbar puncture between January 2012 and January 2014. Data on the presence of microbiological pathogens in CSF, CSF inflammation markers (white blood cell [WBC] counts, protein levels and glucose CSF/serum ratio) and blood inflammatory responses (WBC count, C-reactive protein [CRP], neutrophil percentage) were collected by reviewing patient files.

We included data from 576 infants (median age 12.5 days, interquartile range, 6–27 days) of whom 383 (66.5%) were born prematurely. In total, 16 bacterial pathogens (3.0%) and 21 viruses (5.5%) were detected in CSF. *Escherichia coli* was detected in 5 cases (1.0%), Enterovirus was detected in 12 cases (3.1%). Leucocytosis in CSF was associated with identification of a pathogen in CSF. Increased CRP was associated with the identification of a bacterial pathogen in CSF.

Bacterial or viral pathogens were only identified in a small proportion of infants with a clinically suspected CNS infection. Leucocytosis in CSF was associated with CNS infection in infants. An increased CRP was indicative of bacterial meningitis.

## Abstract 2

Supplemental Digital Content is available in the text

## 1 Introduction

Central nervous system (CNS) infections are associated with high morbidity and mortality.^[1]^ In children with bacterial or viral meningoencephalitis, severe complications ranging from loss of hearing, permanent motor impairment to death are described.^[2–4]^ The development and severity of complications and (long-term) sequelae is related to the causative pathogens and standard level of health care facilities, with high complication rates described in bacterial disease and in lower developed countries.^[5–7]^ In neonates, clinical signs and symptoms of CNS infections are often nonspecific and include fever, hypothermia, food retention, skin lesions, irritability, or general malaise. Blood and cerebrospinal fluid (CSF) analyses are often performed to diagnose or rule out (severe) CNS infections.

Elevated white blood cell (WBC) counts, increased total protein, and a decreased CSF/serum glucose ratio are widely used indicators of CNS infections.^[8]^ Limited data are available describing biochemical CSF characteristics in neonates. Previous studies have focused on defining diagnostic reference values, rather than examining possible predictive relations between biochemical characteristics and causative infectious pathogens.^[9]^ Other studies performed in neonates have focused on describing CSF characteristics with regard to WBC, protein, and glucose levels, rather than establishing a correlation between causative pathogens and CSF characteristics in childhood CNS infections. These studies are therefore not useful in determining the diagnostic value of these commonly used CSF biomarkers.^[10–12]^

C-reactive protein (CRP), WBC count, and neutrophil percentage are commonly used inflammatory biomarkers in blood but there is no consensus about their ability to distinguish between bacterial or viral pathogens.^[11]^ Previous studies have reported a possible correlation between an increased serum CRP level and a bacterial CNS infection.^[13,14]^ No correlation between an increased WBC count or neutrophil percentage and a bacterial infection has previously been reported.^[15]^ No data on the specificity of these blood biomarkers in the context of neonatal CNS infection were available.

We have described microbiological test results and biochemical characteristics in CSF and blood, in infants younger than 90 days with a clinically suspected CNS infection. Furthermore, we have evaluated the use of biochemical characteristics to differentiate between a bacterial or viral CNS infection in infants.

## 2 Materials and methods

### 2.1 Study population

As part of a retrospective cohort study, we included all infants with a clinically suspected CNS infection, who underwent a venous blood and a lumbar puncture between January 2012 and January 2014. Selection was based on the availability of lumbar puncture and microbiological test results, as all infants clinically suspected for a CNS infection will undergo a lumbar puncture. The final diagnosis of meningitis was based on the detection of a causative pathogen in CSF. All children were younger than 90 days when included at 1 of the 3 participating centers (Amsterdam University Medical Centers, location Academic Medical Center (AMC) and Onze Lieve Vrouwe Gasthuis (OLVG) in Amsterdam, and Flevohospital in Almere). The AMC is a tertiary university hospital, whereas the OLVG and Flevohospital are general hospitals in (the vicinity of) the Amsterdam area. Included infants were divided across 3 age groups: infants younger than 28 days (neonates), infants between 29 and 56 days, and infants older than 57 days. We compared term (born after a gestational age of ≥37 weeks) and prematurely born infants (born after a gestational age of ≤37 weeks). Prematurely born infants were divided across three subgroups, using the World Health Organization's definition of prematurity; extremely premature (gestational age <28 weeks), very premature (gestational age, between 28 and 32 weeks) and moderately premature (gestational age, between 32 and 37 weeks).^[16]^

### 2.2 Microbiological processing of CSF and blood samples

The presence of bacterial and/or viral pathogens was assessed at the medical microbiology laboratory of each participating hospital, using routine bacterial CSF cultures and Real time Polymerase chain reaction (RT-PCR) (AMC) and FTC EPA PCR (Flevohospital and OLVG). All samples were specifically tested for the presence of the following viruses: herpes simplex virus, human parechovirus, cytomegalovirus (CMV), varicella zoster virus, and Enterovirus. The same testing protocol was used in all participating hospitals. No changes were made to the testing protocol during the entire study period. A true CNS infection was defined as the presence of a bacterial or viral pathogen in CSF except when both the clinical microbiologist and the clinician considered the pathogen as contaminant as could be the case with, for example, coagulase-negative staphylococci, micrococci, propionibacteria, corynebacteria, or diphteroids. Assessment of bacterial or viral pathogens in CSF, blood or, stool samples, were dependent on data availability.

CSF- and hematological characteristics were assessed at the local biochemical laboratories using standardized methods. All data on microbiological test results, CSF characteristics (WBC count, total protein, and CSF/glucose serum ratio), and blood biochemical analysis (CRP, WBC count, and neutrophil percentage) were collected from patient files.

### 2.3 CSF and blood characteristics

Traumatic lumbar punctures were excluded from the analyses based on unreliability of these results. A traumatic lumbar puncture was defined as an erythrocyte count of >15000 (3/cells/mm^3^) in CSF. We used previously described age-specific cut-off values for WBC count in CSF.^[9]^ A WBC count >57 (3/cells/mm^3^) was considered increased for infants younger than 28 days. The cutoff values used for infants between 29 and 56 days, and infants older than 56 days were respectively 27 (3/cells/mm^3^) and 15 (3/cells/mm^3^). A total protein level of ≥1,32 g/L in CSF was considered increased and CSF/serum glucose ratio of <0,6 was considered decreased. Assessed blood inflammatory markers included CRP, WBC count and neutrophil percentage. An increased CRP level was defined as a CRP value of ≥10 mg/L, a WBC count of ≥20 × 10^9^ cells/L was considered increased, and an increased neutrophil percentage was defined as >70%.

### 2.4 Statistical analysis

SPSS statistical software (version 23.0, SPSS Inc, Chicago IL) was used for statistical analysis. Numerical variables were expressed as mean plus standard deviation (SD) if equally distributed or median and interquartile range (IQR) if data were skewed. Statistical analysis was performed with the non-parametric Mann-Whitney *U* test for WBC count in blood, neutrophil percentage, CRP, WBC count in CSF, total protein in CSF and CSF/serum glucose ratio. The level of statistical significance was *p* < 0.05.

## 3 Results

### 3.1 Study population

We reviewed the medical records of 576 infants <90 days of age (357 from AMC, Amsterdam, 150 from OLVG, Amsterdam and 69 infants from Flevohospital, Almere) of which 347 (60.2%) were males. The median age was 12.5 days (interquartile range [IQR], 6–27 days) including 446 (77.4%) neonates. Two-thirds of the included infants (66.5%, n = 383) were born prematurely. The majority of infants were included during a stay at the Neonatal intensive care unit (NICU) (61.8%, n = 356) (Table 1).

**Table 1:**
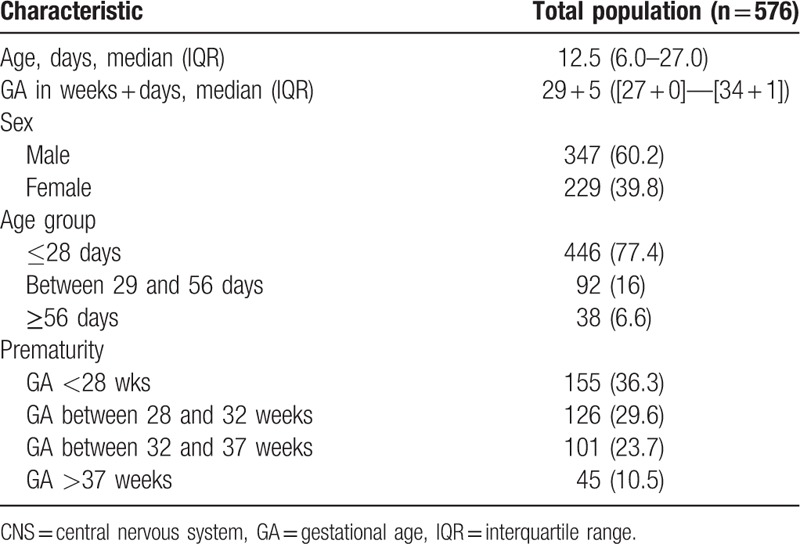
Patient characteristics and demographic distribution. Demographic characteristics of 576 infants presenting with suspected CNS infection between January 2012 and January 2014. Data are displayed as median (IQR) or n (%).

### 3.2 CSF microbiological characteristics

Information on bacterial cultures of CSF was available for 92.3% (n = 525) of the included patients. The presence of a viral pathogen was evaluated in 383 CSF samples (66.8% of included patients) (Table 2 and Addendum Table 4a and 4b). We identified 51 bacterial pathogens in CSF of which 33 samples were considered contaminated and thus excluded for further analyses. A total of 16 (3.0%) CSF samples were considered true bacterial CNS infections. *Escherichia coli* was the most frequently cultured bacterial pathogen (5/525 samples [1.0%]). Two cases of *Streptococcus agalactiae* (HS group B) infections (0.4%) were detected. A viral pathogen was detected in 21 of 383 CSF samples (5.5%), of which enterovirus was detected most frequently (12/383 samples, 3.1%). Human parechoviruses were identified in 3 of 383 CSF samples (0.8%). One infant had a coinfection with *Escherichia coli* (*E coli*) and CMV. *Toxoplasma gondii* was detected in 2 (0.4%) cases. A blood culture was performed in 469 neonates, of which 146 (31.1%) tested positive for the presence of a bacterial pathogen. We detected 5 (of 469) HS group B infections (1.1%). Two cases of HS group B infections were found in CSF. The majority of positive blood bacterial cultures were considered contaminated. *Staphylococcus epidermidis* was the most prevalent bacterial pathogen in blood cultures (39/146 samples, 26.7%), followed by *Staphylococcus capitis* (17/146 samples, 11.6%), and multiple undifferentiated Staphylococci species (16/146 samples, 11.0%). We found that (93.8%) of the proven bacterial CSF infections correlated with a positive bacterial blood culture (15/16 samples). In these cases, the same isolated pathogen was identified in both the CSF and blood culture. A fecal culture or PCR was only performed in 4 cases, in which no pathogens were identified.

**Table 2:**
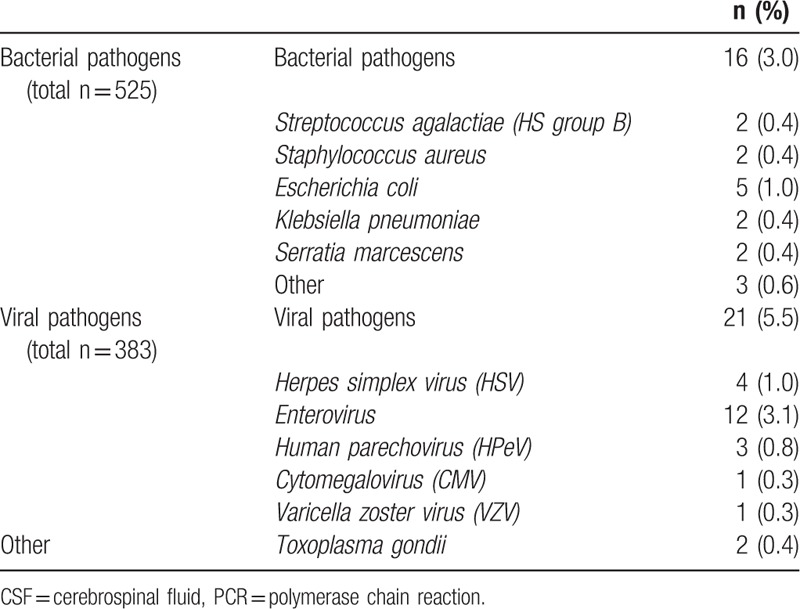
Microbiological CSF results. This table shows the micro-organisms that were identified in CSF, by the use of either a bacterial/viral culture or PCR. Data are displayed as n (%).

### 3.3 Biochemical characteristics in CSF and blood

The median WBC count was 12 measured as 3/cells/mm^3^ (IQR, 5.0–33.0) in CSF samples, with an increased WBC count detected in 59 of 446 samples (13.2%). When a virus was detected, elevated WBC levels were found in 4 of 21 cases (19.0%). The median total protein was 0.95 g/L (IQR, 0.58–1.39), and increased total protein levels were found in 128 of the 438 samples (29.2%). A decreased glucose CSF/blood ratio was found in 119 of 258 samples (20.7%), with a range of 0.62 (IQR, 0.45–0.75) (Table 3 and Addendum Table 4a and 4b).

**Table 3:**
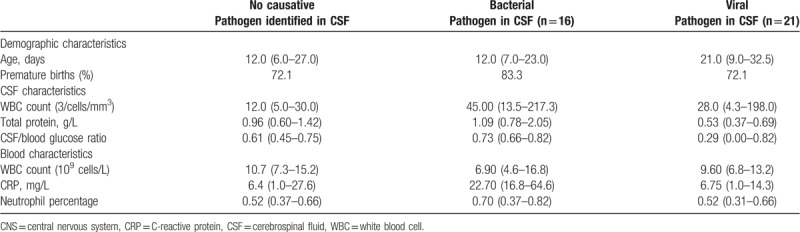
CSF and blood characteristics in proven bacterial or viral infection in CSF. This table shows the differences in descriptive values for different CSF and blood parameters in neonates with a proven CNS infection versus children without a CNS infection. All data are displayed as median (IQR).

An elevated WBC count in CSF was significantly associated with a proven CNS infection in CSF (increased WBC count/CNS infection [30.8%] vs increased WBC count/no CNS infection (12.2%), *P* = .006). No significant correlation between an increased total protein in CSF, a decreased CSF/serum glucose ratio or any of the chosen hematological inflammation markers was found. There was a significant difference in CRP and increased total protein levels between infants with a positive bacterial compared to a positive viral CSF test result. CRP was significantly higher in neonates with bacterial CNS infection, as compared to those with a viral CNS infection (*P* = .010). Total protein levels were significantly higher in infants with a bacterial as compared to a viral CNS infection (*P* = .003).

The median CRP (milligram per liter) was 6.75 mg/L (IQR, 1.00–27.13) with an increased CRP level in 213 of the 488 tested samples (43.6%). The median blood WBC count (10^9^ cells/L) was 10.90 × 10^9^ cells/L (IQR, 7.35–15.80), with increased WBC counts in 66 of 489 samples (13.5%).

## 4 Discussion

In this retrospective study, epidemiology blood and CSF characteristics in infants suspected of a CNS infection, *E coli*, and enteroviruses were the most frequently identified causative pathogens.

Despite the frequency in which blood and CSF analysis are performed, we were only able to identify a causative pathogen in (6.8%) children with a clinically suspected CNS infection. However, in a large proportion of included infants, viral pathogens were not evaluated.

Higher numbers of *E coli* infections have previously been reported; this difference may be explained by the use of different definitions of CNS infection.^[17,18]^ We detected a lower incidence of HS group B infections in CSF and blood, than previous studies.^[19–21]^ The predominance of *E coli* as bacterial pathogen in our study might be explained by the high amount of prematurely born infants included in our study, as higher incidence numbers of *E coli* infections in prematurely born infants have previously been reported.^[22,23]^ Another explanation for a lower incidence of HS group B infections may be caused by the change of prophylactic antibiotic use for maternal HS group B carriership.

Higher incidence numbers of enteroviral neonatal CNS infections (ranging from 15% to 49%), as compared to our study were previously reported.^[24,25]^ This difference may be explained by a difference in study population. In contrast to previous research, this study only evaluated infants <90 days instead of all ages. However, an underestimation of viral pathogens may have occurred, as viral diagnostics were performed in only a small proportion of included infants.

As in previous literature, we identified an increased WBC count in CSF as a significant marker of CNS infections in neonates. However, no consensus on the predictive value of an increased WBC count, when distinguishing between different pathogens in CNS infections could be identified in the available literature. One study reported on a correlation between an increased WBC count and the presence of an enterovirus.^[17]^ A result not specifically shown in our data, nor replicated in several other studies.^[15,18,26]^ In line with previous research, we were unable to differentiate between a bacterial or viral CNS infection using WBC count or CSF/serum glucose ratio.^[18,26,27]^ However, we did find significantly higher total protein levels associated with the identification of a bacterial pathogen, compared viral pathogens.

Furthermore, we have shown significantly higher CRP levels associated with the identification of a bacterial pathogen. This corresponds to previously conducted studies that have reported on a correlation between bacterial CNS infections and increased CRP levels.^[13,14]^

The lack of conclusive data on the value of CSF biomarkers as predictors of CNS infections in neonates has been contributed to the use of a wide variability of cutoff values, when assessing CSF inflammatory markers, in infants younger than 90 days.^[17,28]^

The identification of a true infection is furthermore challenged by the lack of a uniform distinction between contamination and CNS infection. This is especially challenging for cases in which commensal bacteria were identified in CSF.^[29]^ Diagnostic interpretation might have differed across different hospitals and settings. A difference in patient population might have influenced clinical decision-making and subsequent treatment of patients. This is especially true when comparing term and prematurely born infants.

In this study, we collected data on a large population of infants <90 days of age with a clinical suspicion of CNS infection across different hospitals in the Netherlands, combining both bacterial and viral and biochemical characteristics associated with CNS infections in neonates. Despite these strengths several limitations need to be addressed. Our study was limited because of the retrospective set-up, which allowed us to only analyze data on available diagnostic test results. As elaborate testing is not routinely performed, not all biochemical CSF data were available for every individual infant, as a result, only general increase and decrease trends could be shown.

The evaluation of clinical symptoms and CNS infections fell outside the scope of this study and no data on clinical symptoms were collected. Therefore, no analysis of a possible correlation between clinical symptoms and causative pathogens was performed, this remains something for future research to assess.

Furthermore, this study was conducted in a single tertiary hospital and 2 affiliated centers. Generalizability of study results may therefore be limited.

We have shown that a microbiological pathogen was detected in a small proportion of neonates with a suspected CNS infection. The possibility of a CNS infection increases if WBC count is increased. Bacterial CNS infection is more likely in the presence of an increased CRP and an increased total protein in CSF, as opposed to viral CNS infections.

## Author contributions

**Supervision:** Andrea Bruning, Dasja Pajkrt.

**Writing – original draft:** Dirkje de Blauw, Andrea Bruning, Dasja Pajkrt, Linde Vijn.

**Writing – review & editing:** Joanne Wildenbeest, Katja Wolthers, Maarten Biezeveld, Anne-Marie van Wermeskerken, Femke Nauta.

## Supplementary Material

Supplemental Digital Content
