# Supplementary material for: Blood and cerebrospinal fluid characteristics in neonates with a suspected central nervous system infection
Source: Medicine (Baltimore). 2019 Jun 21;98(25):e16079. doi: 10.1097/MD.0000000000016079 (PMC6636939; doi:10.1097/MD.0000000000016079)
Supplement: Supplemental Digital Content [file medi-98-e16079-s001.docx]

**Addendum Table 4a. Infection markers for different bacterial infections.** This table shows the individual values for C- reactive protein (CRP) and white blood cell (WBC) count in blood and cerebrospinal fluid (CSF), as well as total protein in CSF for the identified bacterial infections.

| **Pathogen** | **Blood** | | **CSF** | | **Traumatic puncture** |
| --- | --- | --- | --- | --- | --- |
|  | **CRP**  **(mg/L)** | **WBC (10^9/L)** | **WBC**  **(3/cells/mm3)** | **Total protein**  **(g/L)** |  |
| **Bacterial infection** |  | | | |  |
| #1 *Escherichia coli* | 10 | 24.3 | 651 | 1.64 | Yes |
| #2 *Escherichia coli* | 21 | 16.1 | 159 | 1.34 | No |
| #3 *Escherichia coli* | 33 | 5.2 | 15 | 1.22 | No |
| #4 *Escherichia coli* | - | - | - | 8.64 | No |
| #5 *Escherichia coli* | 24 | 5.7 | 7668 | 4.16 | No |
| *#1 Streptococcus agalactiae* | 21 | 17.9 | 392 | 0.82 | No |
| *#2 Streptococcus agalactiae* | 4 | 2.7 | 2 | 0.74 | No |
| *#1 Staphylococcus aureus* | 21 | 16.4 | 42 | 0.79 | No |
| *#2 Staphylococcus aureus* | 293 | 10.6 | 150 | 2.63 | Yes |
| *#1 Klebsiella pneumoniae* | 0 | 17.5 | 246 | 2.46 | Yes |
| *#2 Klebsiella pneumoniae* | 57 | 14.2 | 72 | 1.63 | Yes |
| *#1 Serratia marcescens* | 3 | 2.2 | 45 | 1.07 | No |
| *#2 Serratia marcescens* | 115 | 7.2 | 63 | - | No |
| *#1 Enterobacter aerogenes* | 55 | 20.3 | 45 | 1.10 | No |
| *#1 Enterobacter cloacae* | 4 | 22.4 | 126 | 1.44 | Yes |
| *#1 Streptococcus species* | 13 | 11.3 | 5 | 0.47 | No |

Abbreviations: CSF= cerebrospinal fluid; WBC =white blood cell count; CRP=C-reactive protein. A traumatic puncture is defined as an erythrocyte cell count of > 15000 3/cells/mm3 in CSF

**Addendum Table 4b. Infection markers for different viral infections.** This table shows the individual values for C- reactive protein (CRP) and white blood cell (WBC) count in blood and cerebrospinal fluid (CSF), as well as total protein in CSF for the identified viral infections.

| **Pathogen** | **Blood** | | **CSF** | | **Traumatic puncture** |
| --- | --- | --- | --- | --- | --- |
|  | **CRP**  **(mg/L)** | **WBC (10^9/L)** | **WBC**  **(3/cells/mm3)** | **Total protein**  **(g/L)** |  |
| **Viral infection** |  | | | |  |
| *#1 Herpes simplex virus* | 7 | 7.0 | 36 | 0.58 | No |
| *#2 Herpes simplex virus* | 1 | 26.3 | 24 | 1.38 | No |
| *#3 Herpes simplex virus* | 2 | 13.7 | 57 | - | No |
| *#4 Herpes simplex virus* | 0 | 9.7 | - | - | No |
| *#1 Enterovirus* | - | - | 306 | 1.35 | No |
| *#2 Enterovirus* | 14 | 9.5 | 1 | 0.37 | No |
| *#3 Enterovirus* | 13 | 6.8 | 31 | 0.69 | No |
| *#4 Enterovirus* | 0 | 9.9 | - | - | No |
| *#5 Enterovirus* | 1 | 9.8 | 245 | 0.67 | No |
| *#6 Enterovirus* | 134 | 22.8 | 11 | 0.29 | No |
| *#7 Enterovirus* | 15 | 4.4 | 2 | 0.58 | No |
| *#8 Enterovirus* | 4 | 7.9 | 26 | 0.39 | No |
| *#9 Enterovirus* | 19 | 5.6 | 2 | 0.53 | No |
| *#10 Enterovirus* | 12 | 7.4 | 4 | 0.36 | No |
| *#11 Enterovirus* | 1 | 13.0 | 907 | 0.89 | No |
| *#12 Enterovirus* | 9 | 10.1 | 275 | 0.43 | No |
| *#1 Human parechovirus* | 7 | 6.6 | 5 | 0.38 | No |
| *#2 Human parechovirus* | 253 | 18.5 | 30 | 0.02 | No |
| *#3 Human parechovirus* | 0 | 4.1 | - | - | No |
| *#1 Cytomegalovirus* | - | - | - | 8.64 | No |
| *#1 Varicella zoster virus* | - | - | - | - | No |

Abbreviations: CSF= cerebrospinal fluid; WBC =white blood cell count; CRP=C-reactive protein. A traumatic puncture is defined as an erythrocyte cell count of > 15000 3/cells/mm3 in CSF
